# Supplementary material for: Short-term costs and cost-efficiency of HPV triage strategies in a high HIV-prevalence setting: Evidence from Botswana
Source: PLoS One. 2025 Sep 8;20(9):e0328803. doi: 10.1371/journal.pone.0328803 (PMC12416716; doi:10.1371/journal.pone.0328803)
Supplement: S1 File — This file contains: S1 Table. Cost parameters collected through micro-costing (2022 USD). S1 Figure: Post-hoc analysis of total costs of hrHP screening followed by three triage strategies, including screening and treatment costs, by modelled increasing treatment costs. S2 Table. Post-hoc analysis with varied short-term costs of treatment of both true and false positive cases of CIN2 + detected using three triage strategies following HPV positivity. Costs do not reflect long term treatment or delayed treatment and health outcomes for true cases missed. S3a: Demographic characteristics for those with and without pathology, among participants living with HIV. S3b: Demographic characteristics for those with and without pathology, among women without HIV. (DOCX) [file pone.0328803.s001.docx]

**Supplemental Information**

| **S1 Table**. **Cost parameters collected through micro-costing (2022 USD)** | | | |
| --- | --- | --- | --- |
| **Parameters** | **Base** | **Low Estimate** | **High Estimate** |
| **Primary hrHPV screen** |  |  |  |
| **Personnel** |  |  |  |
| Counseling | $0.66 | $0.23 | $10.00 |
| Specimen instructions/label sample | $0.45 | $0.34 | $7.00 |
| Explain results process | $0.23 | $0.11 | $3.00 |
| Paperwork | $0.47 | $0.11 | $0.91 |
| Lab processing | $0.74 | $0.55 | $1.11 |
| Results to patient | $0.45 | $0.45 | $0.45 |
| **Supplies** |  |  |  |
| Hand soap | $0.41 | $0.21 | $0.62 |
| Gloves | $0.07 | $0.04 | $0.11 |
| HPV Specimen collection kit | $0.90 | $0.45 | $1.35 |
| HPV Cartridge | $9.00 | $4.50 | $13.50 |
| Cartridge shipping & customs | $2.70 | $1.35 | $4.05 |
| Specimen transport | $0.18 | $0.09 | $0.27 |
| **Capital** | $0 |  |  |
| **VIA** |  |  |  |
| **Counseling** | $0.66 | $0.34 | $1.13 |
| VIA examination | $0.85 | $0.45 | $1.36 |
| VIA+ counseling | $0.43 | $0.11 | $1.02 |
| Patient on/off table | $0.40 | $0.23 | $0.79 |
| Paperwork | $0.47 | $0.11 | $0.34 |
| **Supplies** |  |  |  |
| Hand soap | $0.41 | $0.21 | $0.62 |
| Gloves | $0.07 | $0.04 | $0.11 |
| Acetic acid | $0.23 | $0.12 | $0.35 |
| Methylated spirit | $0.22 | $0.11 | $0.33 |
| Cotton balls | $0.05 | $0.03 | $0.08 |
| Pregnancy test strip (1/20 patients) | $0.04 | $0.02 | $0.06 |
| Water soluble lubricant | $0.05 | $0.03 | $0.08 |
| **Capital** |  |  |  |
| Speculum | $12.90 | $6.45 | $19.35 |
| Gallipots | $7.64 | $3.82 | $11.46 |
| Sponge holding forceps | $29.85 | $14.93 | $44.78 |
| Medicine trolley | $137.90 | $68.95 | $206.85 |
| Examination light | $85.37 | $42.69 | $128.06 |
| **Colposcopy** |  |  |  |
| **Personnel** |  |  |  |
| Counseling | 3.36 | 2.99 | 3.73 |
| Colposcopy exam | 1.96 | 1.49 | 3.36 |
| Counseling (using post LEEP from TAM) | 0.89 | 0.37 | 1.49 |
| Patient on / off table | 1.45 | 0.37 | 2.24 |
| Paperwork | 1.26 | 0.37 | 2.61 |
| **Supplies** |  |  |  |
| Hand soap | $0.41 | $0.21 | $0.62 |
| Gloves | $0.07 | $0.04 | $0.11 |
| Acetic acid | $0.23 | $0.12 | $0.35 |
| Methylated spirit | $0.22 | $0.11 | $0.33 |
| Cotton balls | $0.05 | $0.03 | $0.08 |
| Pregnancy test strip (1/20 patients) | $0.04 | $0.02 | $0.06 |
| Water soluble lubricant | $0.05 | $0.03 | $0.08 |
| **Capital** |  |  |  |
| Speculum, Collin, coated lateral opening | $821.99 | $411.00 | $1,232.99 |
| Gallipots | $7.64 | $0.00 | $0.00 |
| Sponge holding forceps | $29.85 | $3.82 | $11.46 |
| Medicine trolley | $137.90 | $14.93 | $44.78 |
| Examination light | $85.37 | $68.95 | $206.85 |
| Colposcope | $991.76 | $42.69 | $128.06 |
| Notes: Costs have been inflated to 2022, converted to USD, but do not reflect annualization. | | | |

**S1 Figure:** Post-hoc analysis of total costs of hrHP screening followed by three triage strategies, including screening and treatment costs, by modelled increasing treatment costs.

| **S2 Table.** **Post-hoc analysis with varied short-term costs of treatment of both true and false positive cases of CIN2+ detected using three triage strategies following HPV positivity. Costs do not reflect long term treatment or delayed treatment and health outcomes for true cases missed.** | | | | | |
| --- | --- | --- | --- | --- | --- |
| **Triage test Option following primary hrHPV + result** | **True positive** | **False positive** | **Potential short term treatment costs (true+false positive)** | **Total program cost, including treatment** | **ICER** |
|  |  |  |  |  |  |
| ***Base treatment cost: $16*** |  |  |  |  |  |
| VIA | 112 (81-132) | 151 (123-172) | $6,208.00 | $64,049 | - |
| Genotyping | 176 (160-188) | 385 (362-407) | $14,112.00 | $66,554 | $39 |
| Colposcopy | 124 (105-142) | 179 (156-200) | $7,168.00 | $74,813 | dominated |
|  |  |  |  |  |  |
| ***Base treatment cost: $32*** |  |  |  |  |  |
| VIA | 112 (81-132) | 125 (107-143) | $12,416.00 | $70,257 | - |
| Genotyping | 176 (160-188) | 321 (301-321) | $28,224.00 | $80,666 | $163 |
| Colposcopy | 124 (105-142) | 145 (127-168) | $14,336.00 | $81,981 | dominated |
|  |  |  |  |  |  |
| ***Base treatment cost: $64*** |  |  |  |  |  |
| VIA | 112 (81-132) | 276 (235-315) | $24,832.00 | $82,673 | - |
| Colposcopy | 124 (105-142) | 324 (283-368) | $28,672.00 | $96,317 | $1,137 |
| Genotyping | 176 (160-188) | 706 (663-748) | $56,448.00 | $108,890 | $242 |
| Notes: | | | | | |

| **S3a: Demographic characteristics for those with and without pathology, among participants living with HIV** | | | |
| --- | --- | --- | --- |
| Characteristic | Pathology  N=719 | No pathology  N=104 | P |
| Age, (years) | 43.1 ± 9.8 | 41.1 ± 9.9 | 0.053 |
| Education |  |  | 0.38 |
| ≤Primary | 151 (21.0) | 18 (17.3) |  |
| ≥Secondary | 668 (79.0) | 86 (82.7) |  |
| Employed | 414 (57.6) | 52 (50.0) | 0.14 |
| Marital status |  |  | 0.21 |
| Single | 539 (75.0) | 87 (83.7) |  |
| Married | 138 (19.2) | 12 (11.5) |  |
| Divorced / Separated | 10 (1.4) | 2 (1.9) |  |
| Widowed | 32 (4.5) | 3 (2.9) |  |
| Gravidity |  |  | 0.16 |
| 0 | 49 (6.8) | 3 (2.9) |  |
| 1-3 | 456 (63.4) | 63 (60.6) |  |
| ≥4 | 214 (29.8) | 38 (36.5) |  |
| Parity |  |  | 0.12 |
| 0 | 60 (8.3) | 4 (3.8) |  |
| 1-3 | 495 (68.8) | 69 (66.3) |  |
| ≥4 | 164 (22.8) | 31 (29.8) |  |
| Age of sexual debut (years) | 19.1 ± 2.7 | 19.2 ± 2.4 | 0.75 |
| Lifetime sexual partners |  |  | 0.66 |
| 0 | 0 (0.0) | 0 (0.0) |  |
| 1-5 | 424 (59.0) | 60 (57.7) |  |
| ≥6 | 290 (40.3) | 44 (42.3) |  |
| Missing | 5 (0.7) | 0 (0.0) |  |
| Smoking |  |  | 0.11 |
| Current or former | 56 (7.8) | 13 (12.5) |  |
| Never | 663 (92.2) | 91 (87.5) |  |
| Self-reported history of cervical cancer screening | 550 (76.5) | 65 (62.5) | 0.0004 |
| Self-reported history of cervical excisional procedure | 8 (1.1) | 0 (0.0) | 0.28 |
| Duration of HIV diagnosis (years) | 9.6 ± 5.7 | 7.9 ± 5.3 | 0.003 |
| Currently taking ART | 718 (99.9) | 104 (100.0) | 1.0 |
| Length of time on ART (years) | 8.0 ± 5.3 | 6.2 ± 4.6 | 0.0008 |
| CD4 Count (per µL), recent |  |  | 0.40 |
| ≥ 500 | 554 (77.1) | 78 (75.0) |  |
| 350-499 | 99 (13.8) | 13 (12.5) |  |
| 200-349 | 45 (6.3) | 11 (10.6) |  |
| < 200 | 21 (2.9) | 2 (1.9) |  |
| CD4 Count (per µL), nadir |  |  | 0.13 |
| ≥ 500 | 0 (0.0) | 0 (0.0) |  |
| 350-499 | 49 (6.8) | 3 (2.9) |  |
| 200-349 | 355 (49.4) | 63 (60.6) |  |
| < 200 | 314 (43.7) | 38 (36.5) |  |
| Missing | 1 (0.1) | 0 (0.0) |  |
| Detectable viral load (≥ 40 copies per milliliter) | 8 (1.1) | 0 (0.0) | 0.28 |

Data presented as mean ± standard deviation or n (%)

| **S3b: Demographic characteristics for those with and without pathology, among women without HIV** | | | |
| --- | --- | --- | --- |
| Characteristic | Pathology  N=594 | No pathology  N=61 | P |
| Age (years) | 40.3 ± 11.5 | 40.9 ± 13.6 | 0.69 |
| Education |  |  | 0.48 |
| ≤Primary | 96 (16.2) | 12 (19.7) |  |
| ≥Secondary | 498 )83.8) | 49 (80.3) |  |
| Employed | 343 (57.7) | 35 (57.4) | 0.96 |
| Marital status |  |  | 0.42 |
| Single | 415 (69.9) | 47 (77.1) |  |
| Married | 148 (24.9) | 12 (19.7) |  |
| Divorced / Separated | 4 (0.7) | 1 (1.6) |  |
| Widowed | 27 (4.6) | 1 (1.6) |  |
| Gravidity |  |  | 0.80 |
| 0 | 52 (8.8) | 6 (9.8) |  |
| 1-3 | 395 (66.5) | 38 (62.3) |  |
| ≥4 | 147 (24.8) | 17 (27.9) |  |
| Parity |  |  | 0.41 |
| 0 | 60 (10.1) | 7 (11.5) |  |
| 1-3 | 426 (71.7) | 39 (63.9) |  |
| ≥4 | 108 (18.2) | 15 (24.6) |  |
| Age of sexual debut (years) | 19.2 ± 2.4 | 19.5 ± 2.2 | 0.37 |
| Lifetime sexual partners |  |  | 0.01 |
| 0 | 0 (0.0) | 1 (1.6) |  |
| 1-5 | 418 (70.4) | 44 (72.1) |  |
| ≥6 | 170 (28.6) | 16 (26.3) |  |
| Missing | 6 (1.0) | 0 (0.0) |  |
| Smoking |  |  | 0.56 |
| Current or former | 31 (5.2) | 4 (6.6) |  |
| Never | 563 (94.8) | 57 (93.4) |  |
| Self-reported history of cervical cancer screening | 319 (53.7) | 23 (37.7) | 0.02 |
| Self-reported history of cervical excisional procedure | 2 (0.3) | 0 (0.0) | 1.0 |

Data presented as mean ± standard deviation or n (%)
