# Supplementary material for: Short-term costs and cost-efficiency of HPV triage strategies in a high HIV-prevalence setting: Evidence from Botswana
Source: PLoS One. 2025 Sep 8;20(9):e0328803. doi: 10.1371/journal.pone.0328803 (PMC12416716; doi:10.1371/journal.pone.0328803)
Supplement: Attachment — Submitted filename: Response to reviewers_2025Feb4.docx [file pone.0328803.s003.docx]

Editorial Board

PLOS ONE

Dear Academic Editor and Reviewers,

Thank you for reviewing our research article that we have retitled, based on reviewer comments: " Short-term costs and cost-efficiency of HPV triage strategies in a high HIV-prevalence setting: Evidence from Botswana.” We also added a coauthor, Anna Modest, who conducted a sensitivity analysis requested by reviewers. We appreciate the reviewers' thorough assessment and valuable feedback, which have strengthened our paper.
**Minor comments**

1. Please ensure that your manuscript meets PLOS ONE's style requirements, including those for file naming. We have made minor edits to the title and file names.

2. We note that the grant information you provided in the ‘Funding Information’ and ‘Financial Disclosure’ sections do not match. When you resubmit, please ensure that you provide the correct grant numbers for the awards you received for your study in the ‘Funding Information’ section. I am unable to find the ‘Financial Disclosure’ section.

3. We note that you have indicated that there are restrictions to data sharing for this study. PLOS only allows data to be available upon request if there are legal or ethical restrictions on sharing data publicly. The data needed to evaluate the cost analysis are found within the paper’s existing tables, figures, and supplemental information. The data for the parent study are available up on request. We have updated the data availability section.

4. Please include captions for your Supporting Information files at the end of your manuscript, and update any in-text citations to match accordingly. We have added captions at the end of the manuscript.

Additional Editor Comments:

P12 and treatment of dysplasia.5,1“ references not sequential. We checked all the references to ensure they are correct and sequential.

P12 „While hrHPV testing has the highest sensitivity and specificity for detecting cervical dysplasia“ it might be misleading to state hrHPV testing has the highest specificity especially as ref17 acknowledges that cytology had higher specificity. Ref 16 might be outdated (2006). We removed “and specificity” and updated the citation with Chatzistamatiou et al. 2017.

P13 „A prospective cohort study enrolled 3,000 women to evaluate the performance of primary hrHPV DNA testing followed by triage…“ it might be beneficial to specify the reasons why the patients originaly visited the health facilities? General screening population or some higher risk population (almost 50% were hrHPV positive)? We have clarified in the “Study population and setting section” that: “Participation was open to women visiting district health facilities for any service, thus representing the general screening population.”

P14 „participants underwent a speculum examination where 5% acetic acid was applied to the cervix using a cotton swab. VIA…“ for clarity it might be best to introduce the VIA abbreviation at this point. We introduced the abbreviation as suggested.

P16 „In brief, among the 2,969 women screened for HPV, 1,480 were positive and 1,269 had histopathology data for analysis“ the somewhat large discrepancy 1480 vs 1269 might warrant elaboration to exclude potential biases. Thank you, while we believe that 14% is a low lost to follow-up rate, we conducted additional sensitivity analyses to compare the characteristics of women with and without pathology stratified by HIV status. We included additional tables in the supplemental information that compare characteristics of participants with and without pathology stratified by HIV status (S3a and S3b). The only significant differences among participants living with HIV were longer durations of diagnosis and time on ART. Among all participants, those with pathology had an increased likelihood of self-reporting a history of cervical cancer screening. Among those without HIV, there were differences in numbers of sexual partners, but the differences might be driven by 6 participants missing this data. We have included the new analysis in the methods and results under “Sensitivity analyses” and explained the potential biases in the Discussion’s limitations section.

P16 „The health outcome is the number of histologically confirmed cases of CIN2+.“ Consider presenting the total number of identified CIN2+ cases of the 1269 histopathology assessed cases since this is the basis of later calculations. We added the following content to the results: “Outcomes were based on 2,959 women undergoing a primary hrHPV DNA test,1,269 women screening positive for high-risk HPV and completing triage, and 412 confirmed cases of CIN2+.”

P18-20 it might be necessary to emphasize in Table 1, Table 2 and Table 3 that primary screening was done with hrHPV genotyping since this is not the most common screening method. Using another primary hrHPV test would make the triage by 8type restricted HR HPV genotyping another added cost. We have included this information in Tables 1-3.

P20 since the discrepancy between finally examined cases and total HPV positive cases was not explained, it is not completely clear how the missing 211 cases affect calculation of true Cin2+ cases? We added the following content to the limitations: Lost to follow-up occurred among 14% of participants, which resulted in missing pathology results. In our sensitivity analysis, we found limited differences between participants who were and were not missing data. While participants missing pathology were more likely to have a shorter duration of living with HIV and being on ART, previous research has not found a significant difference between HIV and ART duration and increased risk for CIN 2+.(30) However, participants with pathology were more like to self-report prior receipt of cervical cancer screenings than those missing pathology. Thus, if our sample included women less likely to have CIN2+ (because they were more likely to have been screened and treated), the average cost per CIN2+ detected would be biased higher than what would be found in the general population for all strategies.

P20 copy paste errors „personnel (VIA Base: $followed by supplies“. Fixed.

P21 „As all strategies included the cost of hrHPV DNA testing..“ testing should be replaced by genotyping to avoid misleading the readers. This also applies to other places within the manuscript where the reader might be mislead that any hrHPV primary testing would have the same outcomes/costs. We have clarified throughout and also added content to the limitations: “however, these results will not be generalizable to hrHPV triage strategies where genotyping is not provided alongside the primary HPV result.”

**Reviewer Comments to the Author**

**Reviewer #1**: Authors Wynn et al present a costing study comparing three methods for HPV triage (8-type genotyping, VIA and colposcopy) in Botswana. hrHPV testing with 8-type genotyping is presented as the best strategy for triaging HPV positive women; a finding which is importantly consistent irrespective of HIV status. Overall, I found the collection and reporting of costs to be thorough and clear.

I have suggested minor revisions for this paper, and my suggested revisions are thus:

(1) I suggest re-branding this outcome as an "efficiency measure" rather than an ICER, and also removing the claim that this is a cost-effectiveness study from the title. We clarified that the outcome is a measure of efficiency and updated the title.

(2) While it is transparent in the discussion that only costs for primary screening and triaging are considered (rather than total screening pathway costs), it is my opinion that more discussion about the possible impact that this would have on the findings is warranted. Screening approaches with low specificity (such as screen-and-treat with HPV and hrHPV with 8-genotype triaging) are likely to appear less expensive (relative to how expensive they currently look compared to high-specificity approaches) once repeat HPV testing of triage-negative women (and any subsequent precancer treatment) is considered. This is likely to be even more significant for settings with high HIV prevalence, as women living with HIV are more likely to be carrying a persistent HPV infection than HIV-negative women. We have added the following content to the discussion:

“While our study included the costs of primary hrHPV screening and three strategies for triaging women who were hrHPV positive, we did not collect costs associated with the subsequent treatment and rescreening cascade. In practice, the next steps following an 8-type hrHPV genotype restriction would vary by context. For example, higher-resource settings may opt for routine biopsies to determine treatment needs, while others may rely on VIA. As identified in the parent study,(6) VIA and colposcopy have limitations, particularly in detecting endocervical lesions. Women with HIV may have increased inflammation and non-HPV-related cervicitis, making visible lesions more common and potentially larger, leading to higher VIA positivity rates.(34-36) These findings highlight the need for tailored triage strategies that consider both resource availability and patient-specific factors such as HIV status.”
(3) A minor comment but please check ref 19 in the introduction. The referenced paper is very old and doesn't seem to support your statement. We have updated the reference.

(4) Suggest including the country (Botswana) in the title. We added Botswana to the title

**Reviewer #2: It’s my pleasure to have the opportunity to review the manuscript. I have gone through the manuscript in detail and please see my comments below.**

Abstract
1. The authors stated that the analysis was conducted among women with and without HIV. However, the results did not mention any stratification regarding the findings. It could be better to state upfront that results are consistent in women with and without HIV. We have updated the results section in the abstract to say: “Results were consistent in women with and without HIV.”

2. The results stated that “personnel and hrHPV test kit cartridge costs as significant drivers of overall costs.” We updated the abstract conclusion to say, “We found that 8-type genotype restriction was optimal compared to hrHPV screening combined with VIA or colposcopy. Cost estimates can inform future studies that examine the long-term costs and health outcomes of HPV-based two-stage screening algorithms.”

3. The difference between “long-term” vs. “short-term” is not well defined in the abstract.
Introduction. We clarified that post-hoc analysis incorporated the “average treatment costs for precancer.” We also updated the Post-hoc analysis paragraph in the methods (page 8): “As a post-hoc analysis, we incorporated treatment costs among both true and false positive cases of precancer in each strategy using a range of possible treatment costs, including our estimate of LEEP costs and potential average treatment costs found in the literature.”

1. When stating that “hrHPV testing has the potential for rapid scale- up and increased access to screening through self-sampling, making it a highly attractive option for screening in sub-Saharan Africa”, have there been studies assessing the acceptance and performance of self-sampling in the region? Yes. We added two references on acceptability and performance:

Bogale, A. L., Teklehaymanot, T., Ali, J. H., Kassie, G. M., Medhin, G., Baye, A. Y., & Shiferaw, A. Y. (2022). Comparison of self-collected versus clinician collected cervicovaginal specimens for detection of high risk human papillomavirus among HIV infected women in Ethiopia. BMC women's health, 22(1), 360. https://doi.org/10.1186/s12905-022-01944-2

Elliott T, Kohler RE, Monare B, Moshashane N, Ramontshonyana K, Muthoga C, Wynn A, Howett R, Luckett R, Morroni C, Ramogola-Masire D. Performance of vaginal self-sampling for human papillomavirus testing among women living with HIV in Botswana. Int J STD AIDS. 2019 Oct;30(12):1169-1176.

2. hrHPV test usually has a lower specificity compared to cytology.
Koliopoulos G, Nyaga VN, Santesso N, Bryant A, Martin‐Hirsch PP, Mustafa RA, Schünemann H, Paraskevaidis E, Arbyn M. Cytology versus HPV testing for cervical

Thank you. We edited the sentence so as not to state that hrHPV has the highest specificity, and we included the suggested citation.

3. Cytology triage is a common triage method for hrHPV-positive results. Could the authors expand on why the test “is not available at a population level”?

We have added the following additional details: “For example, in Botswana, cytology is not deemed logistically feasible by the National Cervical Cancer Prevention Program as a triage strategy due to cost and personnel requirements.(20) Further, previous research showed that cytology had suboptimal performance compared to VIA.(6)”

4. At the end of the introduction, please add that the analysis was conducted among both women with and without HIV. Here is the updated sentence in the last paragraph of the introduction: “Our study was conducted among women living with and without HIV in Botswana and estimates the average per-screening costs of three two-stage screening algorithms that begin with primary hrHPV testing followed by 1) 8-type HPV genotype restriction (16/18/31/33/35/45/52/58), 2) VIA or 3) colposcopy for those who test positive for hrHPV.”

Methods

1. Subtitles have different formats in terms of capitalizing the first letters. Please keep them consistent. Fixed.

2. Please specify which 8 types of HPV were considered eligible to be treated in text and also cite to support the choice of the 8 HPV type to be eligible for treatment. We added the following details to the second paragraph in the “HPV screening and triage strategies” subsection: The first triage strategy was management of women positive for only the 8 HPV genotypes most associated with cervical cancer (HPV 16,18,31,33,35,45,52,58).(3) For this strategy, we assumed that women would be referred for immediate treatment.

3. Is the cost estimated as per screening or per woman who tested hrHPV positive? Are these two equivalent? We updated the study design to confirm that we also costed the primary hrHPV screen: “The main outcomes were the implementation costs of the primary hrHPV screen and the additional cost of three triage strategies among woman who tested hrHPV positive.”

4. The number of procedures observed to determine personnel utilization seems to vary a lot by the triage test (ranging from 6 to 20). Does it depend on the complexity of the procedure? We clarified that, “The number of observations was determined both by staff capacity and complexity of the procedure.”

5. Does recording the number of supplies used during a procedure require “timing”? Why was the “time-and-motion observation” used to assess the number of supplies utilized? While staff were conducting time and motion observations to determine the type and time of personal involved in each activity, they also noted the supplies used. The supplies did not require timing.

6. Was the CHEERS only apply to the post-hoc analysis? If not, it would be better to state it upfront in the “Study design” section. We have moved the CHEERS statement to the study design section.

7. The post-hoc analysis seems to be a crucial portion as it reflects the “short-term” cost of the treatment. It seems more appropriate to include the “post-hoc” in the main analysis and justify the significance of including it in the study. Also, please define “short-term” earlier in the text. We agree that the post-hoc analysis provides important insight into the costs of treatment following hrHPV triage. However, we refer to it as "post-hoc" because estimating the cost of treatment was not included in the original study design and we used different methods to estimate potential treatment costs. Unlike the primary cost analysis, the treatment cost estimates rely on estimates from our study as well as inputs from the literature.

To address your comment, we clarified that treatment costs were estimated separately, and include inputs from the literature. In the discussion we suggest that real-world costs of treatment for precancer and cervical cancer are needed. Further, we clarified in the Method’s subsection on “post-hoc” analysis that treatment costs were limited to precancer treatment (e.g. LEEP).

8. The sensitivity and specificity of the three triage strategies seem to fit in the results section. These results were derived from the parent study.

9. Colposcopy is usually considered the “gold-standard” diagnostic test for detecting CIN2+. It seems that in the current study, colposcopy performance was less ideal and resulted in a lower sensitivity than hrHPV triage, especially in women without HIV. Please see our response to comment #11.

10. It would be better for the readers to understand the context if the authors included a flowchart to describe the screening scheme of the three triage strategies implemented in Botswana. We have noted that the triage scheme can be found in the parent study publication.

11. This is my main question/concern: in real-world settings, when using hrHPV triage, do all women who test positive receive treatment (and what type of treatment would that be), or is a colposcopy still needed to determine the treatment eligibility? If it is the latter, then it’s worrisome, given the colposcopy performance in the current study. In the current study, every hrHPV-positive woman had a histology sample collected to determine their “true health state.” However, in the real world, many true cases of CIN2+ would have been missed if there was “no visible lesion” and not every hrHPV positive was followed to collect a histology sample. We agree. The poor performance of triage testing is the primary reason we are searching for better triage strategies. All hrHPV positive women do NOT receive treatment as standard of care routinely in any context. If all hrHPV+ women were treated, it would result in massive overtreatment – in Botswana for instance, 50% of all women seeking care are HPV+ but <10% have actual pathologic disease (CIN2+). We acknowledge colposcopy is also not perfect - there are many endocervical lesions and visual examination, whether through VIA or colposcopy is not adequate. In high-income countries, women undergo an endocervical curettage when they are referred for a positive screening test (HPV or pap smear) but no lesion on colposcopy, in order to assess the endocervix. This is logistically challenging in resource constrained settings.

Results
1. Table 3: please specify what the numbers in the parenthesis indicate for the column “True CIN2+ Cases Detected”. Please also add the same range for the costs. Updated.

2. Please also add a column of false-positive results in Table 3 to help understand the specificity of the three triage methods. The numbers of false positive are included in the supplemental Table 2.

3. Please revise the sentence: “For VIA and colposcopy triage, personnel (VIA Base: $followed by supplies were the biggest cost drivers.” Fixed.

4. Please revise the sentence: “We also and found that genotyping became more costly ….” We revised as such: “We found that genotyping became costlier than VIA when treatment costs exceeded $10 and costlier than colposcopy when they exceeded $36.”

5. Could the author please explain the potential reasons that the test sensitivity for VIA and colposcopy seemed to differ in women with and without HIV?

Women with HIV likely have more inflammation and non-HPV related cervicitis that contributes to a higher likelihood of seeing visible lesions, as well as having larger HPV-related lesions that are more visible on the ectocervix. Our findings are consistent with other studies showing higher rates of VIA positivity among women with HIV:

Namale G, Mayanja Y, Kamacooko O, Bagiire D, Ssali A, Seeley J, Newton R, Kamali A. Visual inspection with acetic acid (VIA) positivity among female sex workers: a cross-sectional study highlighting one-year experiences in early detection of pre-cancerous and cancerous cervical lesions in Kampala, Uganda. Infect Agent Cancer. 2021 May 11;16(1):31.

Geda YF, Lamiso YY, Berhe TM, Mohammed SJ, Chibsa SE, Endalew DA, Mossa KA, Abeje S, Hussen MA, Gesesse MM. Visual Inspection with Acetic Acid Positivity in Screening and Early Detection of Cervical Dysplasia in Africa, 2023: A Meta-Analysis. Ethiop J Health Sci. 2024 Jan;34(1):3-14.

Tachiwenyika E, Dhodho M, Muchedzi A, Sibanda TP, Mupanguri C, Mukungunugwa S, Mhangara M, Ganje N, Tapera T, Samushonga T, Muzondo M, Dube S, Tafuma T, Chingombe B, Maravanyika A, Nyamundaya T. Prevalence of Cervical Cancer and Clinical Management of Women Screened positive using visual inspection with acetic acid and Cervicography in selected public sector health facilities of Manicaland and Midlands provinces of Zimbabwe, 2021. PLoS One. 2023 Nov 29;18(11):e0294115.

Discussion

1. Please include some comments on the differential performance of the VIA and colposcopy regarding patient’s HIV status. Please see below.

2. Please discuss the implementation of the hrHPV triage in real-world settings (with or without a diagnostic colposcopy). We have added the following content to the discussion section:

While our study included the costs of primary hrHPV screening and three strategies for triaging women who were hrHPV positive, we did not collect costs associated with the subsequent treatment and rescreening cascade. In practice, the next steps following an 8-type hrHPV genotype restriction would vary by context. For example, higher-resource settings may opt for routine biopsies to determine treatment needs, while others may rely on VIA. As identified in the parent study,(6) VIA and colposcopy have limitations, particularly in detecting endocervical lesions. Women with HIV may have increased inflammation and non-HPV-related cervicitis, making visible lesions more common and potentially larger, leading to higher VIA positivity rates.(34-36) These findings highlight the need for tailored triage strategies that consider both resource availability and patient-specific factors such as HIV status.

Sincerely,

Adriane Wynn
