# Supplementary material for: Short-term costs and cost-efficiency of HPV triage strategies in a high HIV-prevalence setting: Evidence from Botswana
Source: PLoS One. 2025 Sep 8;20(9):e0328803. doi: 10.1371/journal.pone.0328803 (PMC12416716; doi:10.1371/journal.pone.0328803)
Supplement: Attachment — Submitted filename: Response to reviewers_2025June1.docx [file pone.0328803.s004.docx]

Editorial Board

PLOS ONE

Dear Academic Editor and Reviewers,

Thank you for continuing to review our research article entitled, "Short-term costs and cost-efficiency of HPV triage strategies in a high HIV-prevalence setting: Evidence from Botswana.” Please see our responses to each comment below.

**Minor**

- p12 wrong reference and or reference format (superscript number 7) “(WLHIV) in Botswana.(6) 7” **Fixed**
- p13 wrong reference and or reference format (superscript number 28) “Economic Evaluation Reporting Standards (CHEERS).(25)28” **Fixed**
- p17 typo “The health outcomes have been previously reported(7)” no space **Fixed**
- p18 “based on reviewer comments,” obsolete **Removed text**
- p18 typo “positive cases of precancerin” **Fixed**
- p20 wrong reference and or reference format (superscript number 8) “and 412 confirmed cases of CIN2+. 8” **Updated**
- p21 it is not necessary to list all 3 panels for the in text Figure 1 callout “(Figure 1. One-way sensitivity analysis of: 1) Primary hrHPV screen, which includes the genotyping triage strategy, 2) Additional cost of VIA as a triage screen, 3) Additional cost of colposcopy as a triage screen.)**” We removed the titles**
- p21 typo “personnelfollowed” **Fixed**
- p22 in text Figure 2 callout doesn’t need to contain the title **We removed the title**
- p24 typo missing fullstop “primary HPV result In this” **Fixed**
- p29 Figure 1 title of panel 1 typo “hHPV”, x-axis title is cropped out “(genotyping is no…” **Fixed**
- p30 Figure 2 y axix title has typo “fof” **Fixed**

**Editor Comments**

While the study undeniably adds interesting cost data and findings, one major concern was not addressed, which when dealing with patient oriented studies, is critical. The chosen triage strategy assumes immediate treatment and in this situation specificity of 30% (commented upon in the review process) cannot be ignored or remain unmentioned in the discussion section. The authors do state in the results section 'We found that genotyping became costlier than VIA when treatment costs exceeded $10 and costlier than colposcopy when they exceeded $36.'. This finding must be discussed in the context of significant overtreatment costs of the false positive cases even if other risks of overtreatment are ignored. We agree and have added content on overtreatment costs in our response to Reviewer 2 below.

**Reviewer 2**

One of my previous comments raised my question on why colposcopy, usually considered the “gold standard” for detecting lesions, was less ideal and had lower sensitivity than hrHPV triage. The authors mentioned the challenge of collecting endocervical curettage without a visible lesion, which is understandable. However, the high sensitivity of the hrHPV triage in the current study is conditioned on a universal biopsy collection regardless of whether a lesion is visible during a colposcopy. Although hrHPV genotyping triage has a high sensitivity, as it will identify more women with true disease, if the diagnostic procedure itself has low performance, we still face the dilemma of the next steps after a positive genotyping triage result. If the next step requires a diagnostic test (VIA or colposcopy) to ensure treatment, the performance of the tests is less ideal as many true cases will still be missed; if the next step is immediate treatment, we suffer from the low specificity (30% as mentioned in the manuscript) and overtreatment. The authors will need to address the limitations of the colposcopy (or other diagnostic procedure) performance. Otherwise, the findings from the current study and the benefit of the high sensitivity of the hrHPV genotyping triage may not be utilized in real-world settings.

Thank you for highlighting the implications of test performance beyond initial screening and triage. We agree that the utility of hrHPV genotyping as a triage strategy hinges on the performance of subsequent diagnostic or treatment procedures, and that weak performance at these later stages can diminish the value of high initial sensitivity. To address this, we have added the following underlined content to paragraph five of the discussion:

While our study included the costs of primary hrHPV screening and three strategies for triaging women who were hrHPV positive, we did not collect costs associated with the subsequent treatment and rescreening cascade. Compared to VIA and colposcopy triage, hrHPV genotyping more accurately narrows the pool of people who need further evaluation; however, the impact of this strategy is dependent on the subsequent diagnostic and/or treatment procedures. For example, if the diagnostic procedure is VIA, many true cases will still be missed. VIA and colposcopy have limitations, particularly in detecting endocervical lesions. Women with HIV may have increased inflammation and non-HPV-related cervicitis, making visible lesions more common and potentially larger, leading to higher VIA positivity rates.(31-33) Further, if the next step is immediate treatment (e.g. LEEP or thermal ablation), overtreatment will occur. This weakness is partially demonstrated by our post-hoc analysis that found hrHPV genotyping became costlier than VIA and colposcopy at relatively low treatment costs due to higher sensitivity and lower specificity. In long-term mathematical modeling studies, preventing the costs of cancer care can balance out the costs of overtreatment.(30) However, the harms caused by overtreatment are not well measured, there is no widely accepted threshold on the maximum number of cases of overtreatment that are acceptable in order to prevent an additional case of cervical cancer, and more research is needed.(34) In practice, the next steps following an 8-type hrHPV genotype restriction would vary by context. For example, higher-resource settings may opt for routine biopsies to determine treatment needs, while others may rely on VIA These findings highlight the need for tailored triage strategies that consider both resource availability and patient-specific factors such as HIV status.

Sincerely,

Adriane Wynn

Assistant Professor

Division of Infectious Diseases and Global Public Health

University of California, San Diego
